# Mr. Shoolbred's Letter to Dr. Jenner, on Vaccination

**Published:** 1805-06-01

**Authors:** John Ring

**Affiliations:** New Street, Hanover Square


### *To the Editors of the Medical and Physical Journal.*

Gentlemen,

THE following Extract of a Letter from Mr. Shoolbred to Dr. Jenner, dated Calcutta, June 21, 1804, cannot fail to be interesting to your readers.

"Notwithstanding the encouragement afforded by Government, and the zeal of individuals, we shall still have many obstacles to encounter, and great opposition to overcome, before the practice of Vaccination can be rendered general in Bengal. But in the other parts of Hindostan, where inoculation for the small-pox was not previously used, and where, consequently, there exists no class interested in prejudicing the natives against it, we may reasonably expect it to spread more rapidly.

"If my report had been two days longer in coming from the press, I should have had the satisfaction of announcing a circumstance that cannot fail to have great weight in its favor, over an extensive region in India; which is, that our late enemy, now our ally, Dowlut Row Scindia, the Chief of the Mahratta Empire, has had one of his children, some say his only child, vaccinated at Ongien; an example which, we may naturally hope, will be soon generally followed throughout his large and populous dominions.

"This sensible act of the head of the Mahrattas, who are all Hindoos of high cast, shews in a very strong point of view, that it is on no essential point of religion the Hindoos of Bengal object to the new practice; but merely from prejudice instilled into their minds by the interest-ed ed inoculating Bramins, founded on some indistinct notion of the disease coming from the cow; they know not in what manner, but, as they profess, in a way which renders it impure to them, and consequently unfit for their adoption. I am not without hopes, however, that we shall in time win over to our side a few of the principal men amongst these inoculators; and thus prevail upon some of the higher class of Hindoos, in Calcutta, to have their children vaccinated; after which we shall have less difficulty in extending its benefits to the middling and lower classes of the natives."

---

In addition to the cases of small-pox, occurring in those who are supposed to have had the disease, I beg leave to add the following.

The first was communicated to me by Mr. Swain, Surgeon, of Mitcham. Miss Worsfold, of Epsom Court, was inoculated by the late Mr. Prowton, of Tower Street; who assured her that she was safe from future infection. Three years after, she caught the disease of her sister, and died of it.

Mrs. Thompson, No. 10, Burleigh Street, Strand, had a disorder, supposed to be the small-pox, at South Moulton, in Devonshire; and was attended by a gentleman of considerable reputation, who entertained no doubt of the nature of the disease. She was afterwards repeatedly exposed to the infection by her mother, and frequently lay on the bed, and sat on the lap of those who had the small-pox; but still resisted infection. After coming to London, she caught the small-pox; and had it in the confluent way, of which the most evident marks remain.

By a letter from Mr. Bell, Surgeon, of Wigton in Cumberland, I received the following information. A child of Mr. Graves was inoculated for the small-pox when a year old. His arm inflamed, but no pustules appeared on the body. Mr. Bell inoculated him a second time, together with a younger child, his sister. His arm inflamed for a few days; while in his sister's arm a pustule rose, and appeared to run through its usual course. These two children have since had the small-pox; one of them in a severe manner, attended with delirium. Another child, who had been vaccinated, associated with them, and slept with them during the whole course of the disorder.

A few days ago, the Earl of Westmeath called on Dr. Jenner, and informed him, that one of his children, who had been inoculated for the small-pox in Ireland two years ago, ago, by an eminent inoculator, who had declared him safe, was then labouring under the confluent small-pox. His lordship told Dr. Jenner, that he thought it his duty to acquaint him with the circumstance; and Dr. Jenner obtained permission for me to see the case.

I saw the child with Dr. Jenner and the Reverend Mr. Jenner. We found him a miserable spectacle, covered with the small-pox from head to foot; and the fetor emitted by the pustules was almost intolerable.

I have also lately seen two cases, one at Clapham, the other in Edgware Road, in which persons supposed to have undergone vaccination, had the small-pox. These cases I went to see by desire of Mr. Forbes and Mr. Griffiths, by whom the patients were vaccinated. Whether the operations were perfectly successful, or whether imperfect, it is impossible for me to say; but the small-pox appearing in a very mild form, I am rather inclined to suspect the latter. Otherwise it must be allowed, that they are exceptions to the general rule; and that the cow-pock is not always a preventive of the small-pox.

I have also seen three other cases, one in Potier's Place, Bermondsey Road; another in Stangate Street, Lambeth; and another in Clement's Passage, Clare Market; in which the small-pox occurred after supposed vaccination. If the accounts of the parents are to be depended on, no genuine cow-pock was produced in these cases; but either spurious pocks, or mere suppurations, followed by yellow or light brown scabs, not leaving proper characteristic indentations.

In the Medical and Chirurgical Review for May is a case of supposed small-pox after vaccination; in which it is stated, that the progress of inoculation was regular; and that the cicatrices were as strongly marked as usual. This, however, is what I must deny. The mother assured me, in the presence of Mr. Burnet, and again since that time, that the scabs which remained after the pocks were not of a dark colour, but of a light brown; and Dr. Croft, when he called to inform me of the case, told me that the scars were rather superficial; which, on examination, I found to be true.

The other child in the same family had deeper scars, and proved far less susceptible of the small-pox. She was vaccinated at the Small-pox Hospital two years after her brother, when a little more caution was observed in taking matter. As she slept with her brother, when covered with the disease, it may easily be supposed that many, many, if not all of her pustules, were *local,* from the application of variolous matter; and that the constitutional symptoms were the result.

Whether the case of Mary Hart, which occurred in 1803, and was not equally investigated, was the small-pox or not, it is impossible now to determine. If it was, and if five hundred more of those who were inoculated at the Small-pox Hospital, with matter taken at a late period, and sometimes from under a dry scab, have since had the small-pox, it cannot excite the least surprise, nor ought it to produce any other effect, but to inculcate more caution for the future.

The next is a case of a child, at No. 13, Stacey Street, Soho, vaccinated by me, lately put to the test of variolous inoculation by Mr. Powel, of Manchester Street. That the process of vaccination was complete no one can doubt, since matter was taken from this subject by Dr. Jenner, to inoculate the child of a nobleman; but Mr. Powell was mistaken in the result of his experiment, from supposing that when a patient is put to the variolous test, no constitutional indisposition should take place.

The contrary, however, is well known; as proofs of which, I need only mention the cases which occurred in the practice of Mr. Goldson and Dr. Rollo. The same thing occasionally takes places in those who are put to the same test after the small-pox.

In the present instance, there were two local pustules in the arms, but no eruption appeared in their neighbourhood; contrary to what usually happens in those who have not previously had either the small-pox or the cow-pock. The pustules on the arm were attended with a considerable itching, and the child scratched them at a very early period; which, his mother informs me, occasioned Mr. Powell to say, that it would prevent them from rising. This is a symptom commonly attendant on those cases, where patients are put to the test.

Mr. Powell himself informs me, that on the eighth day the head of one of the pustules was off, in consequence of scratching, and the inflammation increased. This is precisely what commonly has happened in such cases, particularly at Portsmouth, Woolwich, and Harrow. Hence it is easy to account for pustules in other parts, in consequence of a second inoculation with the nails.

Mr. Powell inoculated a child from one of the pustules on the arm, and produced small-pox. The same might have been done from his own arm, had he made himself the the subject of the test. On the eleventh day two eruptions appeared; one on his leg, the other on his groin. This is no wonder, since, independent of the cause already assigned, there were two others, either of which might have produced twenty such eruptions as occurred in this case. First, the child had constantly slept with the other during variolous inoculation; and, secondly, his mother handled them both by turns.

The only secondary pustule which yielded matter, was that on the leg; which may well be supposed to have originated from the insertion of matter on Friday night, when the primary pustule was ruptured, since it appeared on the third day, and yielded matter on the fourth. It is necessary to remind those who are not very conversant in this subject; that in such as have previously had the small-pox or cow-pock, the pustule excited by inoculation is more early and rapid, than in such as have not had either disorder. Dr. Squirrell recommended that the child should be thrown into a good sweat, in order to produce a plentiful crop, but his advice was not followed.

One eruption appeared on the lip, and three under it; constituting about half the number that was discovered. These, with one or two on the forehead, appeared on the twelfth day, and turned on the fourteenth.

If we allowed that this child had the small-pox after the cow-pock, we must also allow that his mother had the small-pox a second time; for she had two variolous pustules, one on her breast, and the other on her chin, from nursing the child who was under inoculation of the small-pox. The pustule on her chin was larger than any one of those on her son; although her skin may be supposed to be less irritable than that of so young a subject.

Mr. Powell observes, that on the seventh day from the eruption of those on the leg and groin, and the fifth of those in the face, the pustules had totally disappeared; leaving only very *small, dark, horny scabs.* It is worthy of notice, that the pustule on the leg appeared before those on the face, contrary to what generally happens, when the pustules are the effect of fever. We have therefore great reason to believe, that the only real variolous pustule was the effect of inoculation or contact.

This case was seen by Mr. Serjeant, of Prince's Street, Cavendish Square; Mr. Leese, of East Street; and Mr. Fernandez, of Lamb's Conduit Street; who can testify the truth of the principal facts here detailed.

I am, &c.

